# ERK activating peptide, AES16-2M promotes wound healing through accelerating migration of keratinocytes

**DOI:** 10.1038/s41598-018-32851-y

**Published:** 2018-09-26

**Authors:** Sora Lee, Myun Soo Kim, Su-Jin Jung, Daejin Kim, Hyun Jeong Park, Daeho Cho

**Affiliations:** 1 Institute of Convergence Science, Korea University, Anam-ro 145, Seongbuk-ku, Seoul 02841 Republic of Korea; 2 Nano-Bio resources Centre, Sookmyung Women’s University, Cheongpa-ro 47-gil 100 (Cheongpa-dong 2ga), Yongsan-gu, Seoul 04310 Republic of Korea; 3 Department of Anatomy, Inje University College of Medicine, Busan, 47392 Republic of Korea; 4 Department of Dermatology, Yeouido St. Mary’s Hospital, The Catholic University of Korea, Seoul, 07345 Republic of Korea

## Abstract

Wound healing is an important issue that influences quality of life, and the need for products associated with wound healing is growing annually. New materials and therapies for skin wounds are being continuously researched and developed in order to increase treatment efficacy. Here, we show that the peptide AES16-2M comprised of five short amino acid sequences (REGRT) demonstrates efficacy in wound healing. AES16-2M exerted more effective healing than the control in an acute wound model, and tissue regeneration was similar to that of normal tissue in AES16-2M-treated skin. We found that the increase in re-epithelialization by AES16-2M early in wound development was due to migration of keratinocytes; a scratch assay using a human keratinocyte cell line (HaCaT) also demonstrated effective wound closure by AES16-2M. The migration of keratinocytes effected by AES16-2M was promoted through ERK phosphorylation and blocked with U0126, an ERK inhibitor. Moreover, AES16-2M treatment stimulated human dermal fibroblast (HDF) migration as well as keratinocyte. Taken together, these results suggest that AES16-2M can be an effective therapeutic agent for wound healing by promoting migration of keratinocytes and fibroblasts via ERK phosphorylation.

## Introduction

Skin wound healing is a complex and interactive process that involves many factors such as various cell types, cytokines, and growth factors^1–3^. Re-epithelialization is the process by which the skin barrier is re-established through restoration of the damaged epidermis. This process involves the migration of keratinocytes adjacent to the wound and proliferation to support the migrating epithelial tongue^1,4^. After the wound is covered, the epithelium is regenerated through differentiation into the multi-layered epidermis and reconstruction of the basement membrane^1^. As unsuccessful re-epithelialization leads to persistent infections and chronic wounds, timely acute wound healing remains an important health problem^5–7^.

Keratinocytes and dermal fibroblasts play an important role in skin structure formation and maintenance of homeostasis, including skin barrier construction and extracellular matrix (ECM) production^1^. Above all, the migration of keratinocytes is the starting point for the re-epithelialization process, covering the open wound at an early stage^1,4^. Keratinocytes at the edge of the wound are stimulated by growth factors and cytokines such as epidermal growth factor (EGF), transforming growth factor-beta (TGF-β), and platelet-derived growth factor (PDGF) in order to cause their migration along the wound bed^2,3^. Moreover, a series of events such as flattening and lengthening of keratinocytes to the direction of the wound and weakening of cell-cell or cell-matrix adhesion also affects keratinocyte migration. After cell movement is completed, the basement membrane is reconstructed, and the proliferation and differentiation of keratinocytes are resumed in order to complete epidermal regeneration^1^. Fibroblast migration is also affected by growth factors and cytokines, and migrated fibroblasts at the wound site generate and rearrange the ECM fibres, including collagen and elastin^1–3^.

Mitogen-activated protein kinase (MAPK) signalling is extensively involved in cell migration and proliferation regulation^8–10^. Activation of the MAPK signalling pathway, particularly extracellular signal-regulated kinase (ERK) 1/2, is a major regulator of the migration of various cell types^8,10–12^. According to several studies, ERK pathway inhibitors PD98059 and U0126 blocked migration activated via various factors, including fibronectin, collagen, fibroblast growth factor (FGF), and EGF in endothelial cells and fibroblasts^13–15^. In addition, the dominant-negative mutant of mitogen-activated protein kinase kinase (MEK) 1, upstream of ERK, inhibits cell migration by fibronectin and urokinase-type plasminogen activator (uPA) in fibrosarcoma cells^14,16^, while MEK1 activity promotes cell migration in FG carcinoma cells^12,17^. ERK/MAPK signalling is also activated by skin damage, and ERK activation has a direct effect on keratinocyte migration in *in vitro* models. Importantly, down-regulation of this signal significantly reduces cell migration and proliferation, showing critical defects in skin damage repair^17–19^. Thus, the ERK/MAPK pathway plays a pivotal role in the regulation of skin cell migration.

Growth factors, for instance EGF and PDGF, have been widely used as wound healing agents^20,21^. However, various side effects of treatment with high concentrations of growth factors have been reported, including excessive growth of cells, psoriasis, and impaired skin functions^22–24^. Many polymer drugs also have problems, such as not being biodegradable^20,25^. In order to overcome these disadvantages, many peptides have been recently studied^20,25^. In general, peptides are effective molecules with selective signalling such as G-protein-coupled receptors (GPCRs) or ion channel binding. Furthermore, due to their relatively low molecular weight and biodegradable materials, peptides (1) do not persist in the body for a long period of time, (2) are very safe, (3) have low production complexity, and (4) are relatively low-cost^26^.

In this study, we developed a peptide comprised of five amino acids, AES16-2M. AES16-2M showed significant healing effects in animal wound models. In addition, treatment with AES16-2M promoted migration of human keratinocytes (HaCaT) through ERK phosphorylation, and these migration effects were suppressed by the ERK inhibitor U0126. AES16-2M also enhanced migration of human dermal fibroblasts (HDF).

## Results

### AES16-2M accelerates skin wound healing *in vivo* via increasing keratinocyte migration

**Figure 1:**
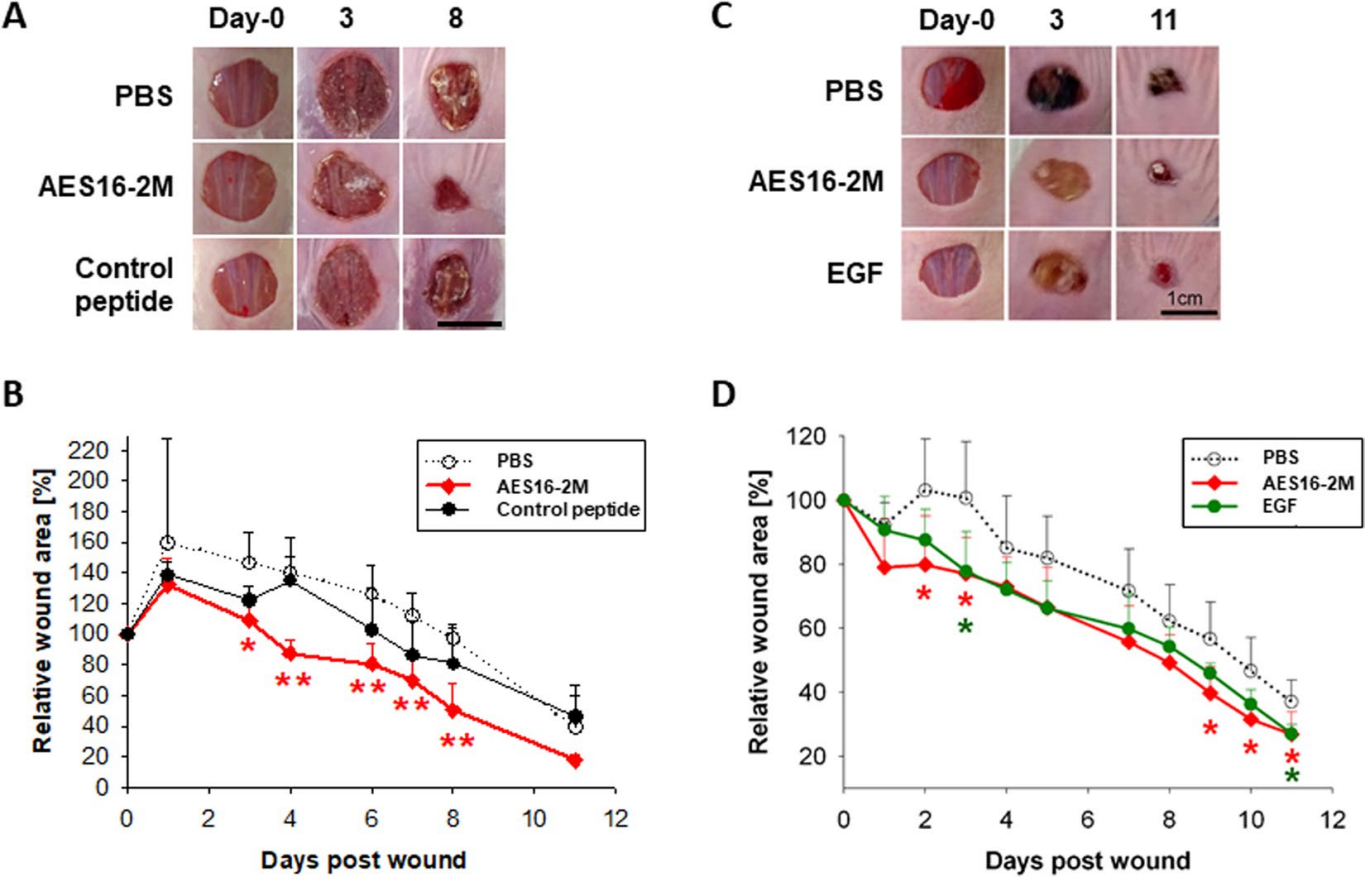
AES16-2M treatment accelerated wound healing in an *in vivo* model. Full-thickness wounds were created on the back of BALB/c wild-type (**A**,**B**) and BALB/c-nude (**C**,**D**) mice. Each group was treated with 50 μl Pluronic® F-127 gel containing either PBS, AES16-2M, control peptide, or EGF. (**A**) A photographic record of wound healing was processed on days 0, 3, and 8 post-injury. Scale bar: 1 cm. (**B**) The residual wound area was calculated as the ratio of the relative wound area to the original wound area on day 0 post-injury (*n* = 5). Error bars, mean ± SD. *p < 0.05, **p < 0.01 (AES16-2M treatment group versus PBS treatment group), two-tailed student *t* test. (**C**) A photographic record of wound healing was processed on days 0, 3, and 11 post-injury. Scale bar: 1 cm. (**D**) The residual wound area was calculated as the ratio of the relative wound area to the original wound area on day 0 post-injury (*n* = 8). Error bars, mean ± SD. *p < 0.05 (AES16-2M or EGF treatment group versus PBS treatment group), two-tailed student *t* test.

AES16-2M is a short peptide consisting of five amino acids; its general characteristics are shown in Table 1. In order to determine the wound healing effect of AES16-2M, BALB/c wild-type mice with acute wounds were treated with AES16-2M gel and wound size was analysed for 11 days. A scrambled peptide was also prepared and administered under the same conditions, as a control to confirm the specificity of AES16-2M. We found that wound size steadily decreased in the AES16-2M treatment group, with significant differences 2–8 days after injury compared to the control peptide (Fig. 1A,B). Moreover, in BALB/c-nude mice, wound size also decreased in the AES16-2M treatment group and the EGF treatment group (positive control) (Fig. 1C), showing significant differences on 2, 3, and 9 days after injury (Fig. 1D).

**Table 1:** The property of peptide AES16-2M.

Peptide properties	
Sequence	REGRT
Molecular Weight	617.66 g/mol
Iso-electric point	pH10.39-10.9
Net charge at pH 7.0	1.0
Average hydrophilicity	1.7 (Good water solubility)

(www.bachem.com, PepCal.com).

**Figure 2:**
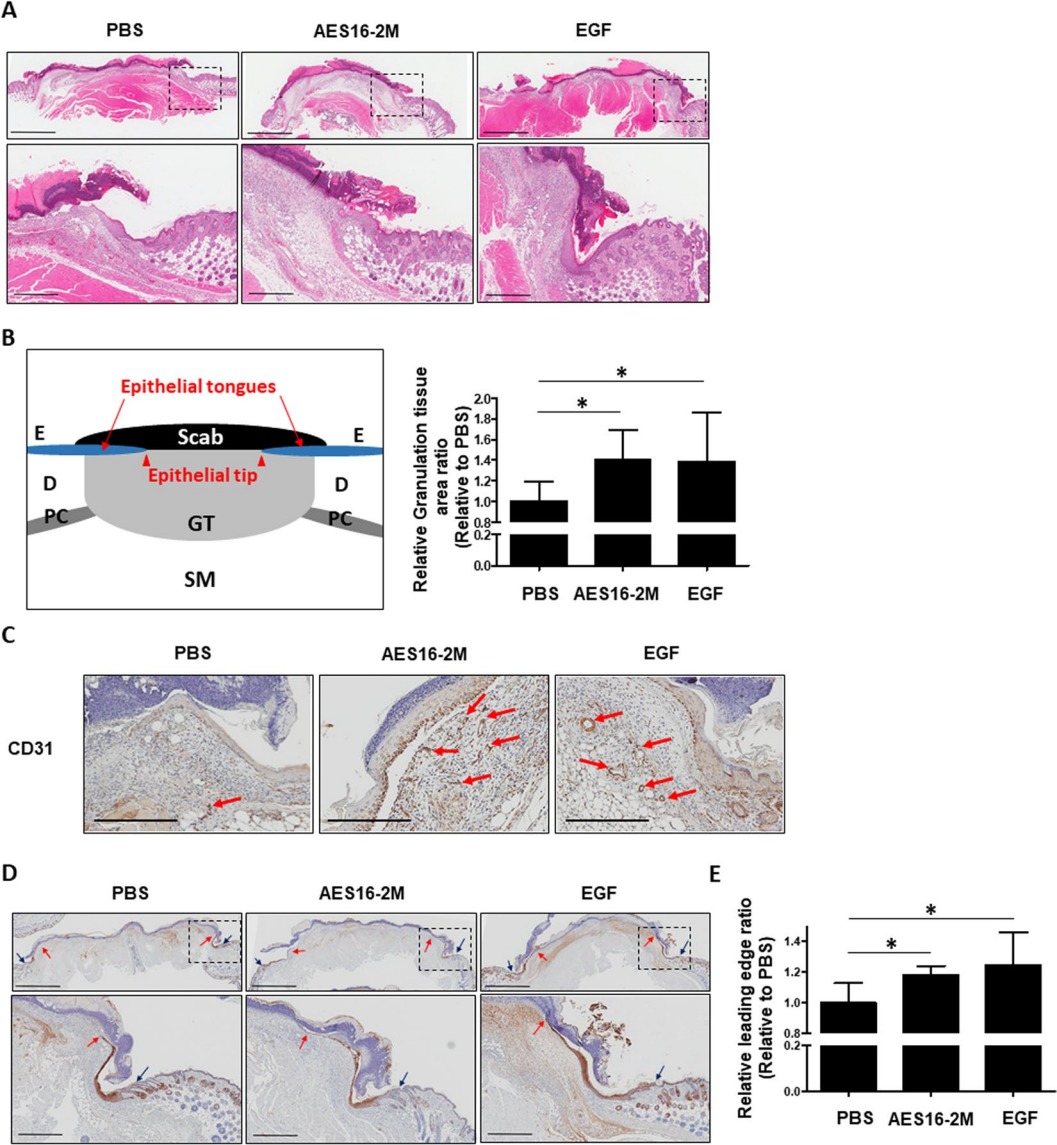
AES16-2M promoted re-epithelialization via keratinocyte migration. On post-wound day 4, serial sections of skin tissue were prepared and examined. (**A**) H&E staining was performed and photographed. (**B**) Schematic representation of wound histology and comparison of granulation tissue area in the AES16-2M treatment group. The area of granulation tissue was measured and graphed based on an H&E-stained section of panel A (*n* = 8). Error bars, mean ± SD. *p < 0.05 (AES16-2M or EGF treatment group versus PBS treatment group), two-tailed student *t* test. (**C**) Immunohistochemistry was performed with anti-CD31 antibody and photographed. Scale bar: 300 μm. (**D**) Slides were stained with anti-keratin 14 antibody and visualized. The blue arrows indicate the wound margin and the red arrows indicate the end of the epithelial tips. (**E**) Measurements of leading-edge ratio in panel D. The leading-edge ratio was calculated as the ratio of the length of the wound to the length of the epithelial tongues (*n* = 6). Error bars, mean ± SD. *p < 0.05 (AES16-2M or EGF treatment group versus PBS treatment group), two-tailed student *t* test. (**A**,**D**) The dashed line in the upper panel is magnified and shown in the lower panel. Upper panel scale bar: 2 mm. Lower panel scale bar: 500 μm.

Next, the wound tissues were collected on day 4 after injury and a histological analysis of the skin wound section was performed using haematoxylin and eosin (H&E) staining (Fig. 2A). We found that in the AES16-2M treatment group, multiple layers of epithelial tissue developed around the wound edge (Fig. 2A) and granulation tissue also increased (Fig. 2B). The formation of granulation tissue in the early stage of a wound is an important step for supporting re-epithelialization and dermal reconstitution^12,27^. Moreover, CD31 expression increased in the AES16-2M treatment group compared to the negative control (Fig. 2C); CD31 is an important measure of dermal restoration, such as angiogenesis^11^. These results demonstrated that the AES16-2M treatment group restored skin tissue similar to the EGF group (Fig. 2A–C). In addition, when the leading-edge ratio of each group was calculated by keratin 14 staining, we found that re-epithelialization was promoted via increasing the migration of keratinocytes by AES16-2M (Fig. 2D); the ratio also increased significantly (Fig. 2E). These data indicate that AES16-2M effectively promoted wound healing through re-epithelialization. These results were very encouraging as AES16-2M had a positive effect with a small amount compared to the positive control, EGF.

### AES16-2M enhances keratinocyte migration *in vitro*

**Figure 3:**
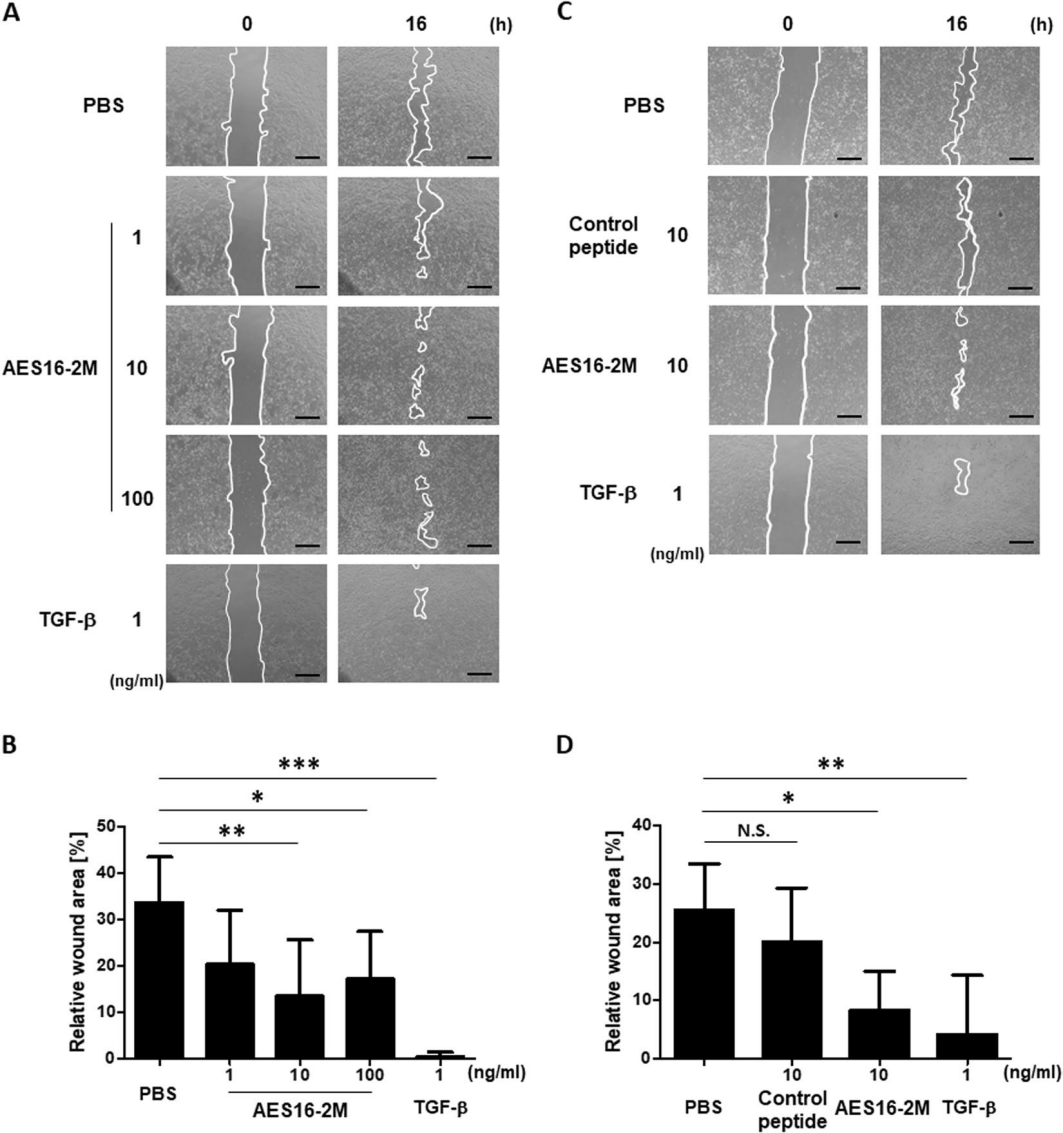
AES16-2M enhanced HaCaT cell migration. HaCaT cells were cultured in 12-well plates. Cells pre-treated with mitomycin C (10 μg/ml) were scratched and incubated with the indicated dose of AES16-2M, control peptide (10 ng/ml), or TGF-β (1 ng/ml). HaCaT cell migration was visualized and recorded using a microscope at 0 and 16 hr post-scratch. The line indicates the border of wound. Scale bar: 500 μm. The residual area of the wound was measured by Image J. Relative wound area was calculated as the ratio of the remaining area at the 16-hr time point to the 0-hr starting point. The six pairs of time points were separately measured and averaged. (**A**) The migration effect according to AES16-2M concentration. (**B**) Graph of measurements in panel A. (**C**) Comparison of the migration effects of the control peptide and AES16-2M. (**D**) Graph of measurements in panel C. Error bars, mean ± SD. *p < 0.05, **p < 0.01, ***p < 0.001 (AES16-2M or TGF-β treatment group versus PBS treatment group). N.S. = No significance (control peptide versus PBS treatment group), one-way ANOVA followed by Tukey post-hoc test.

The migration and proliferation of keratinocytes during wound healing are important steps in re-epithelialization and are used to determine the initial wound healing rate^1,4^. However, we found that the effect of AES15-2M on HaCaT cell proliferation was not significant (Supplementary Fig. S1). In order to next investigate the effect of AES16-2M on cell migration, a scratch wounding assay was performed *in vitro*. The HaCaT cell monolayer was pre-treated with mitomycin C to exclude the proliferative effect. After scratching, AES16-2M was added at the indicated concentrations and the wound area photographed and measured. Image analysis showed that the wound area decreased significantly in the 10 and 100 ng/ml groups (Fig. 3A,B). Unlike AES16-2M, the control peptide had no effect on migration (Fig. 3C,D), indicating that AES16-2M acts specifically to increase migration of keratinocytes.

### ERK phosphorylation mediates the AES16-2M-enhanced migration of keratinocytes

**Figure 4:**
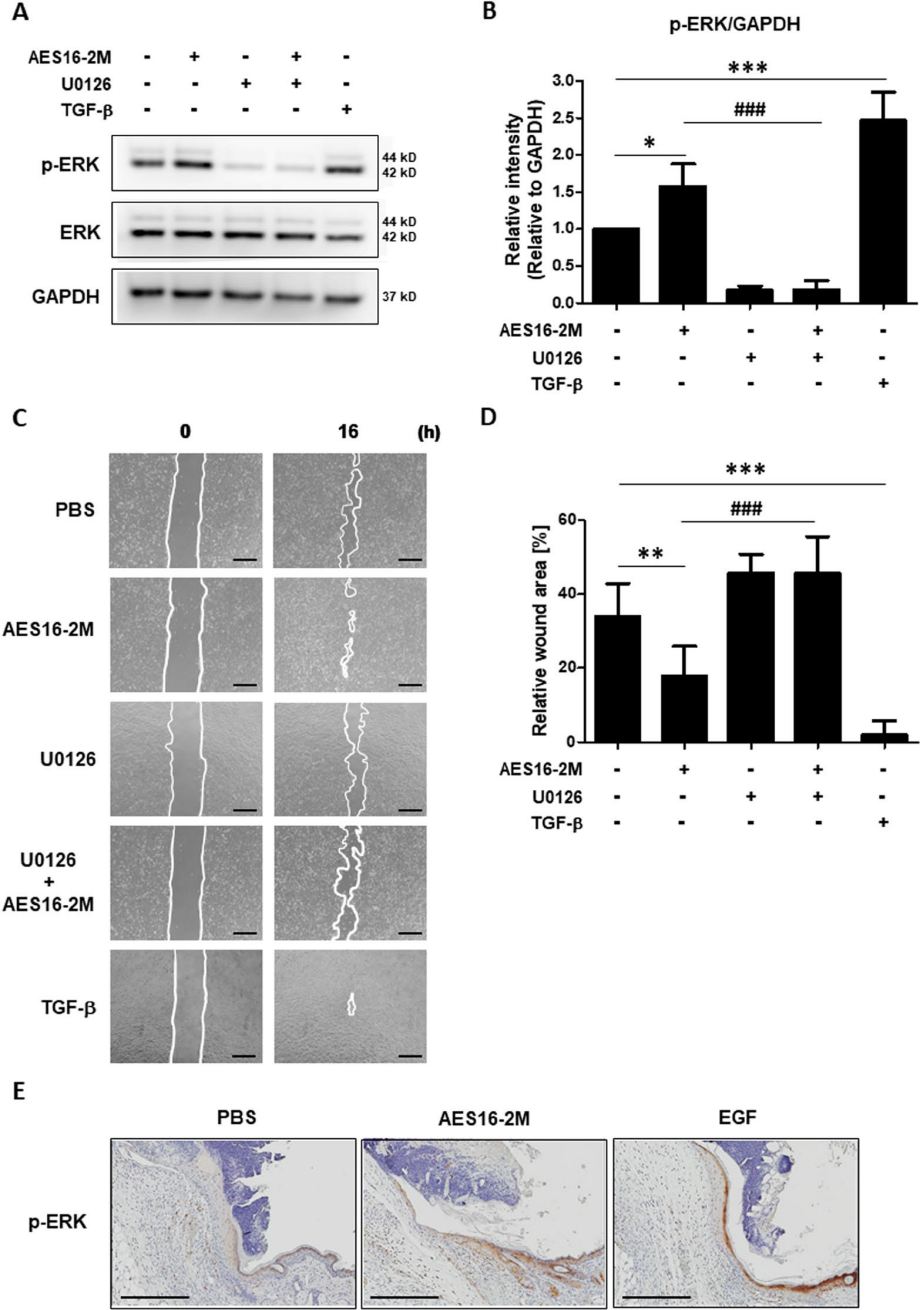
AES16-2M promoted ERK phosphorylation for HaCaT cell migration. (**A**) HaCaT cells were treated with AES16-2M (10 ng/ml), TGF-β (1 ng/ml), and PBS for 30 min under serum-free conditions. The ERK inhibitor (U0126 at 0.1 μM) was added to cells 1 hr prior to treatment, as indicated. Western blotting was performed for phospho-ERK, total-ERK, and GAPDH. The image presented here is a cropped image; full-length blots are presented in Figure S3. (**B**) The band intensity of panel A was measured with Image J and relative density was analysed as the ratio of phospho-ERK to GAPDH (*n* = 3). Error bars, mean ± SD. *p < 0.05, ***p < 0.001 (AES16-2M or TGF-β treatment group versus PBS group), ^###^*p* < 0.001 (AES16-2M versus AES16-2M + U0126 treatment group), one-way ANOVA followed by Tukey post-hoc test. (**C**) For the scratch assay, HaCaT cells were treated with ERK inhibitor (U0126 at 0.1 μM) 1 hr before AES16-2M treatment. Scale bar: 500 μm. (**D**) The residual area of the wound was measured by Image J and the relative wound area was calculated as the ratio of the remaining area to that at 0 hr (*n* = 6). Error bars, mean ± SD. **p < 0.01, ***p < 0.001 (AES16-2M or TGF-β treatment group versus PBS group), ^###^*p* < 0.001 (AES16-2M versus AES16-2M + U0126 treatment group), one-way ANOVA followed by Tukey post-hoc test. (**E**) Immunohistochemistry was performed with anti-phosphorylated (p-)ERK antibody and photographed. Scale bar: 300 μm.

The MAPK signalling pathway is activated upon skin epidermal damage, and down-regulation of ERK signalling inhibits keratinocyte migration^17,18^. Therefore, in order to investigate the effect of AES16-2M on ERK phosphorylation, HaCaT cells were treated with AES16-2M under serum-free conditions and ERK phosphorylation analysed by western blotting. We found that ERK phosphorylation was increased in the AES16-2M group compared to the control group and was blocked in the presence of U0126, an ERK inhibitor (Fig. 4A,B). Furthermore, in the presence of U0126, AES16-2M failed to close the scratch wound of HaCaT cells (Fig. 4C,D). The increase in phosphorylated ERK was confirmed by immunohistochemistry staining of tissue sections (Fig. 4E). These results demonstrate that ERK phosphorylation by AES16-2M is involved in keratinocyte migration.

### AES16-2M induces fibroblast migration

**Figure 5:**
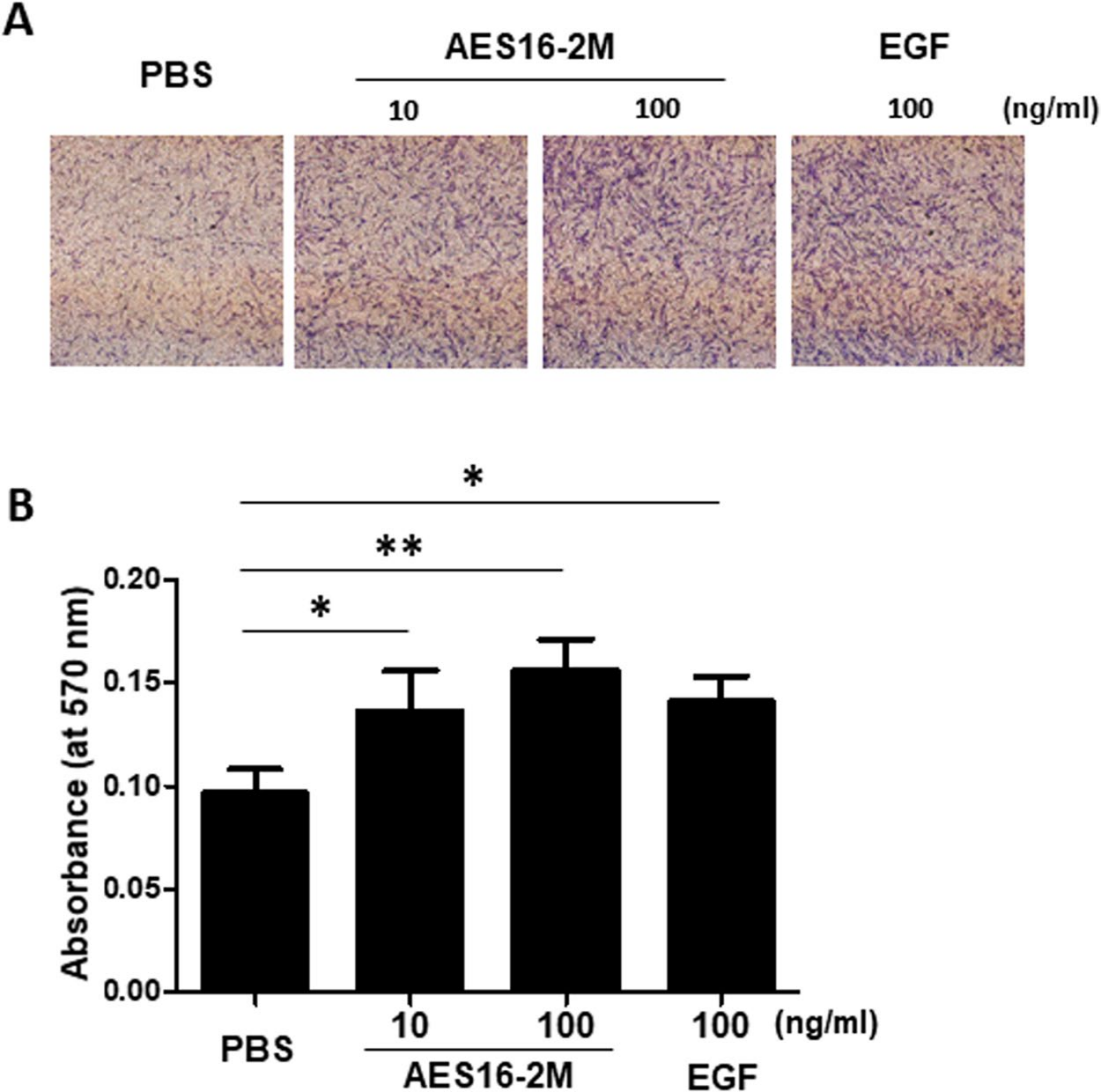
AES16-2M induced HDF cell migration. A transwell migration assay was performed with HDF cells. HDF cells were treated with AES16-2M at the indicated doses or EGF (100 ng/ml) and incubated overnight. HDF cells (1 × 10^4^ cells) were seeded onto a transwell plate and incubated for 24 hr. (**A**) Transwells were stained and recorded by photomicrograph. (**B**) Bar graph showing the eluted density of migrated cells in each group (*n* = 3). The eluted cells were measured by ELISA. Error bars, mean ± SD. *p < 0.05, **p < 0.01 (AES16-2M or TGF-β treatment group versus control group), one-way ANOVA followed by Tukey post-hoc test.

In addition to keratinocytes, the effective migration of fibroblasts to the wound site is also an important determinant of tissue regeneration efficiency^1–3^. In order to determine the effect of AES16-2M on the migration of fibroblasts, a transwell migration assay was performed. HDF cells were treated with AES16-2M overnight and then seeded onto a transwell plate. We found that AES16-2M induced migration of HDF cells in a dose-dependent manner (Fig. 5A,B). Taken together, these results indicate that AES16-2M promotes not only the migration of keratinocytes but also that of fibroblasts.

## Discussion

Rapid keratinocyte migration determines the efficiency of the initial wound healing process^1–3^. The synthesized peptide, AES16-2M, has proven to possess remarkable skin wound healing capabilities through the migration of keratinocytes. AES16-2M showed not only outstanding wound size reduction in animal models, but also tissue reconstruction close to that of normal tissue. In addition, AES16-2M acted to increase keratinocyte and fibroblast migration directly. Moreover, AES16-2M activated the ERK signalling pathway, and when ERK was inhibited with U0126, cell migration of HaCaT decreased. Taken together, these results demonstrate that AES16-2M has an effect on skin wound repair via ERK-mediated stimulation of keratinocyte migration.

The effective healing of damaged skin is a major health problem that must be constantly monitored in specific populations, including elderly individuals, infected patients, and especially individuals with diabetes. As the populations of elderly individuals and diabetic patients grow, the wound healing market grows steadily as well, requiring the development of various materials and treatments. However, the cellular and molecular mechanisms of wound healing are largely unclear^1,6^. In addition, growth factors that are widely used as wound healing agents, including EGF and PDGF^20,21^, have not only disadvantages such as large-scale production cost, but also reports of adverse effects such as tumour formation^23,28^. Therefore, alternative sources for treatment are needed. Peptides are attractive materials for this purpose because of their high efficacy, safety, and predictable metabolism^25,26^. AES16-2M, a small peptide, possesses all of these advantages.

The wound healing process is divided into three stages: inflammation, proliferation, and remodeling^1,2,5^. Re-epithelialization is the proliferation stage, which involves migration and proliferation of keratinocytes and reconstitution of the basement membrane zone (BMZ)^1^. Keratinocyte migration is the first process activated to fill the wound during re-epithelialization^1,29^. In present study, AES16-2M showed a large difference in wound size at early time points in the *in vivo* model (Figs 1 and 2) and also revealed a migration effect in an *in vitro* model (Fig. 3) compared to the controls. Generally, through observation of the shape of keratinocytes via tissue staining at the wound site, keratinocytes can be seen to form a layer at the centre of the wound, as well as several layers near the wound edge^1,29^. According to Laplante AF *et al*., proliferation is active in the posterior section, unlike the leading edge where migration primarily occurs^29^. This proliferation of keratinocytes leads to sufficient cell supply to cover the open wound. However, the proliferation effect of AES16-2M on keratinocytes was insignificant (Supplementary Fig. S1). Several peptides are reported to induce migration but fail to have an effect on proliferation^30,31^. Although proliferation is an important process, the rapid covering of the wound-bed through migration is also required for timely progress to the next step^1,29^. Therefore, co-treatment with AES16-2M and peptides that affect proliferation may be used to improve wound healing, which is an area for further study.

Various signals such as MAPK, phosphoinositide 3-kinase (PI3K), Ras, and protein kinase C (PKC) are involved in cell migration^32,33^. The MAPK pathway has been extensively studied regarding cell migration^33^. According to several studies, Src overexpression promotes keratinocyte migration through ERK activation, and hypoxia reduces expression of CD9 through the p38/MAPK pathway, leading to an increase in keratinocyte migration^34^. However, not all MAPK signals are involved in promoting migration. AES16-2M activated ERK (Fig. 4A), but did not activate p38, and HaCaT cell migration was also not inhibited by treatment with a p38 inhibitor (Supplementary Fig. S2). In several studies, damaged keratinocytes induce matrix metalloproteinase (MMP)−1 expression through ERK activation to promote migration regardless of the p38 or Jun N-terminal kinase (JNK) pathway^35^, and JNK and ERK, but not p38, signalling are required to activinB-induced cell migration^36^. Thus, activation of ERK by AES16-2M is considered to be the primary signal pathway of keratinocyte migration.

In summary, as AES16-2M, consisting of only five amino acids, possesses the advantages of peptides as therapeutic agents and has a wound healing effect via promoting keratinocyte migration, it could be an excellent candidate for a new wound healing agent.

## Materials and Methods

### Ethics statement

The animal study protocol was reviewed and approved by the Animal Experiment Ethics Committee of Sookmyung Women’s University, Republic of Korea (Approval number: SMWU-IACUC-1609-027). All experiments were performed according to approved guidelines.

### Peptide synthesis

AES16-2M (REGRT) and scrambled control peptide were synthesized at PEPTRON (Republic of Korea) in the form of lyophilized powder (1 mg/vial) and analysed by high-performance liquid chromatography (HPLC) analysis to confirm a purity of >98%. The molecular weight of AES16-2M was 618 Da as determined by mass spectrum analysis.

### Cell culture

HaCaT cells were cultured in Dulbecco’s modified Eagle’s medium (DMEM, Welgene, Republic of Korea) containing 10% heat-inactivated foetal bovine serum (FBS, Welgene), 100 U/ml penicillin, and 100 µg/ml streptomycin (Gibco, CA, USA). HDF were obtained from ATCC and cultured in the recommended growth media (ATCC, VA, USA). Cells were grown and maintained at 37 °C in a humidified incubator at 5% CO_2_.

### Mice wound healing experiments

Seven-week-old BALB/c wild-type and BALB/c-nude female mice were purchased from Orient (Republic of Korea) and maintained for 1 week prior to initializing the experiments. In order to create acute wounds, the dorsal skin was shaved, the mice anesthetized with avertin, and a 10-mm-diameter full-thickness wound was punched onto the back using a biopsy punch. Phosphate-buffered saline (PBS), AES16-2M (0.5 μg/wound), or EGF (10 μg/wound, Prospec, Israel) in 50 μl of 20% Pluronic® F-127 gel (Sigma, MO, USA) was applied to the wound area five times. The wound area was photographed and measured by digital planimetry using ImageJ software (National Institutes of Health, MD, USA).

### Cell scratch wounding assay

HaCaT cells (3 × 10^5^ cells/well) were seeded in 12-well plates and cultured as a monolayer to confluence in serum-conditioned medium. After overnight, cells were incubated in serum-free media containing 10 μg/ml mitomycin C (Sigma) for 2 hr to completely inhibit cell proliferation. Scratch wounds were created in confluent monolayers using a P200 disposable micropipette tip. After the suspended cells were removed by washing with serum-free medium, the wounded monolayers were cultured in complete medium with or without AES16-2M. For the inhibitor treatment group, cells were pre-treated with 0.1 µM U0126 (Calbiochem, Germany) 1 hr prior to AES16-2M treatment. Wound area recovery was observed under a phase-contrast microscope and photographed. Using the ImageJ program, the size of the opened area was measured from the digital images. Six randomly selected images were acquired for each group. All experiments were independently carried out in triplicate.

### Transwell migration assay

The fibroblast migration assay was performed using a transwell chamber (Costar, MA, USA) with 6.5-mm-diameter and 8.0 µm-pore polycarbonate filters. Prior to performing the migration assay, cells were treated with or without AES16-2M overnight and then harvested. The lower chamber was filled with 600 μl DMEM supplemented with 10% FBS, and the cells were seeded at a density of 1 × 10^4^ cells in 100 μl serum-free medium in the upper chamber followed by incubation at 37 °C. After 24 hr, the transwell inserts were fixed with methanol, stained with 0.5% crystal violet in 10% ethanol for 10 min, and washed with distilled water (DW). Cells in the upper compartment were removed using a cotton swab and then photographed using light microscopy. For elution, the dyed transwell inserts were dissolved with 10% acetic acid for 20 min and analysed with an enzyme-linked immunosorbent assay (ELISA) microplate reader (Molecular Devices, CA, USA) at 570 nm.

### Immunohistochemistry

Wounded areas surrounded by unwounded skin were dissected on day 4 after injury, fixed in 4% paraformaldehyde, and embedded in paraffin. After slicing into sections, slides were deparaffinized, rehydrated, and H&E staining performed using standard techniques. For immunohistochemistry, antigen retrieval was performed by microwave treatment of the slides for 10 min in citrate buffer (pH 6.0) (Abcam, Cambridge, UK). Slides were blocked with 5% horse serum (Vector Laboratories, CA, USA) in PBS at room temperature for 1 hr and then incubated with rabbit anti-CD31 (1:50; ab28364, Abcam), anti-keratin 14 (1:200; ab181595, Abcam), and anti-phosphorylated (p-)ERK 1/2 (1:100; 4376, Cell Signaling, MA, USA) at 4 °C overnight. After washing, slides were incubated for 30 min with horseradish peroxidase (HRP)-conjugated anti-rabbit antibodies (7074, Cell Signaling) and visualized with 3,3′-diaminobenzidine (DAB) staining (Abcam). Counter-staining was performed with haematoxylin solution (Dako, CA, USA) and slides were then dehydrated and mounted.

### Morphometric analysis of wounds

Morphometric analysis was performed on digital images using the ImageJ program. The extent of re-epithelialization and granulation tissue formation was determined using H&E- and anti-keratin 14 antibody-stained paraffin tissue sections. The length of the epithelial tongue was determined as the distance between the epithelial tip and the margin of the wound. The leading-edge ratio was calculated as the ratio of the length of wound to the length of the epithelial tongues by referring to previous research^37^. Granulation tissue is defined as the tissue matrix, which includes a variety of cell types such as ECM, immune cells, vascular tissue, and fibroblasts^14^. The granulation tissue area was defined as between the wound margins, underneath the neo-epithelium, and above the subcutaneous fat tissue according to previous research^27^.

### Western blot

HaCaT cells were washed twice with PBS and lysed using Pro-prep protein extraction solution supplemented with protease inhibitor mixtures (Intron Biotechnology, Republic of Korea) on ice. Protein extracts were separated by 10% sodium dodecyl sulphate polyacrylamide gel electrophoresis (SDS-PAGE) and transferred to polyvinylidene difluoride (PVDF) membranes (Bio-Rad, CA, USA). The membrane was blocked with 5% non-fat dry milk in Tris-based saline with Tween-20 (TBS-T) for 1 hr and then incubated with rabbit anti-ERK 1/2, anti-p-ERK 1/2 (all at 1:1000; 4695, 9101, Cell Signaling) and anti-GAPDH (1:2000; sc-25778, Santa Cruz, MA, USA) overnight at 4 °C. After washing, the membranes were incubated for 1 hr with appropriate HRP-conjugated secondary antibodies (1:2000; 7074, Cell Signaling) at room temperature for 1 hr. The protein-antibody complexes were detected using an ECL reagent on the ChemiDoc imaging system (Fuji, Japan). The images were quantified with the ImageJ program.

### Statistical analysis

All experiments were performed independently at least three times. Data are expressed as means ± SD. The comparisons between the control and treated groups were ascertained using a two-tailed student *t* test and comparisons among groups were determined using one-way analysis of variance (ANOVA) followed by Tukey post-hoc test. A *p* value < 0.05 was considered statistically significant. Statistical analysis was performed using GraphPad PriSM version 5 for Windows (GraphPad Software, CA, USA).

## Electronic supplementary material

Supplementary Information
