# Supplementary material for: ERK activating peptide, AES16-2M promotes wound healing through accelerating migration of keratinocytes
Source: Sci Rep. 2018 Sep 26;8:14398. doi: 10.1038/s41598-018-32851-y (PMC6158248; doi:10.1038/s41598-018-32851-y)
Supplement: Supplementary file 1 — Supplementary Information [file 41598_2018_32851_MOESM1_ESM.pdf]

ERK activating peptide, AES16-2M promotes wound healing through accelerating migration of keratinocytes.

Sora Lee[1,*], Myun Soo Kim[1,*], Su-Jin Jung[2], Daejin Kim[3], Hyun Jeong Park[4,+], and Daeho Cho[1,+]

[1]Institute of Convergence Science, Korea University, Anam-ro 145, Seongbuk-ku, Seoul 02841, Republic of Korea

[2]Nano-Bio resources Centre, Sookmyung Women's University, Cheongpa-ro 47-gil 100 (Cheongpa-dong 2ga), Yongsan-gu, Seoul 04310, Republic of Korea

[3]Department of Anatomy, Inje University College of Medicine, Busan 47392, Korea

[4]Department of Dermatology, Yeouido St. Mary's Hospital, The Catholic University of Korea, Seoul, Republic of Korea

[*]These authors contributed equally to this work.

[+]Corresponding authors

Daeho Cho Tel.: +82-02-3290-4015; Fax: +82-02-928-8273; E-mail: cdhkor1@gmail.com

Hyun Jeong Park Tel.: +82-2-3799-1230; Fax: +82-2-783-7604; E-mail: hjpark@catholic.ac.kr

Supplementary Materials and Methods

Cell proliferation assays.

HaCaT cells (3×10^3 cells/well, 200 μl) were plated into a 96-well plate. After an overnight incubation, media was removed and cells were treated with 200 μl fresh media that included AES16-2M or EGF. Every 24 hr, for 72 hr, 10 μl Cell Counting Kit-8 (CCK-8) (Dojindo, Japan) was added to each well and the plate was incubated for 3 hr at 37°C. Absorbance at 450 nm was measured with an ELISA microplate reader.

Chemicals and antibodies.

The p38 inhibitor, SB203580, was purchased from Calbiochem. Rabbit anti-p38, anti-phosphorylated (p-)p38 (all at 1:1000; 9212, 9211) were purchased from Cell Signaling.

Supplementary figure legends

Figure S1. AES16-2M had an insignificant effect on HaCaT cell proliferation.

HaCaT cells (3×10^3 cells/well) were cultured in 96-well plates with the indicated doses of AES16-2M or EGF (100 ng/ml). Cell proliferation was detected with CCK-8 analysis at 24, 48, and 72 hr. The relative proliferation rate was calculated as the ratio of the AES16-2M or EGF group versus the PBS group at each time point. (n=5) Error bars, mean ± SD. **p<0.05 (EGF treatment group versus control group), one-way ANOVA followed by Tukey post-hoc test.

Figure S2. HaCaT cell migration induced by AES16-2M was poorly associated with p38 activation.

(A) HaCaT cells were treated with AES16-2M (10 ng/ml), TGF-β (1 ng/ml), and PBS for 30min under serum-free conditions. The p38 inhibitor (SB203580 at 10 μM) was added to cells 2 hr prior to treatment. Western blotting for phospho-p38, total-p38, and GAPDH was performed. The figure is a cropped image; full-length blots are presented in Figure S4. (B) The band intensity of panel A was measured with Image J and relative density was analysed as the ratio of phospho-p38 to GAPDH (n=3). Error bars, mean ± SD. *p<0.05 (TGF-β or SB203580 treatment group versus control group), one-way ANOVA followed by Tukey post-hoc test. N.S.=No significance (AES16-2M versus AES16-2M + SB203580 treatment group). (C) For the scratch assay, HaCaT cells were treated with p38 inhibitor (SB203580 at 10 μM) 2 hr before AES16-2M treatment. Scale bar: 500 μm. (D) The residual area of the wound was measured by Image J and the relative wound area was calculated as the ratio of the remaining area to that

at 0 hr (*n*=6). Error bars, mean ± SD. $**p<0.01$, $***p<0.001$ (AES16-2M, AES16-2M + SB203580 or TGF-β treatment group versus control group). N.S.=No significance (AES16-2M versus AES16-2M + SB203580 treatment group), one-way ANOVA followed by Tukey post-hoc test.

Figure S3. Full-length blots for Figure 4A.

All dashed lines indicate the area cropped and used in the figures. The number displayed is the size of the marker (kD). (A) phospho (p)-ERK. (B) ERK. (C) GAPDH. Blots B and C were performed following stripping the membrane shown in panel A. The arrow indicated non-specific binding (bands remaining after stripping ERK from the membrane). (D) Digitized image for maker detection.

Figure S4. Full-length blots for Figure S2A.

All dashed lines indicate the area cropped and used in the figures. The number displayed is the size of the marker (kD). (A) phospho-p38. The arrow indicated non-specific binding. (B) p38. Blot B was performed following stripping the membrane shown in panel A. (C) Digitized image for marker detection of panels A and B. (D) GAPDH. (E) Digitized image for marker detection of panel D.

Figure S1

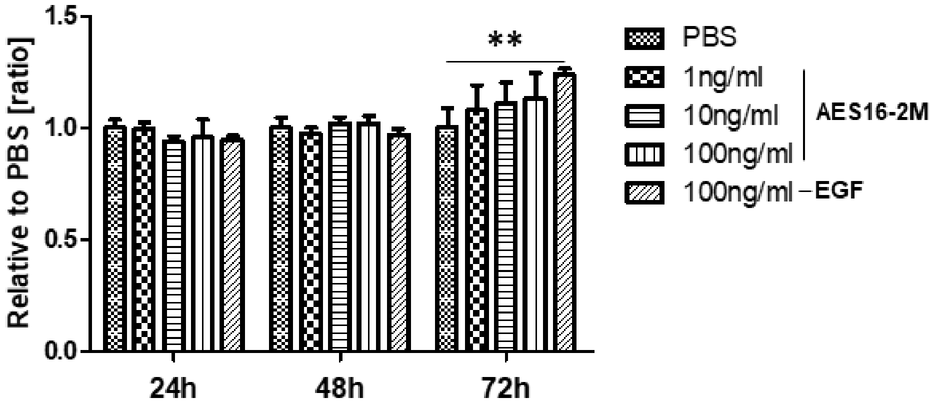
1.5
1.0
0.5
0.0
Relative to PBS [ratio]
**
24h
48h
72h
PBS
1ng/ml
10ng/ml
100ng/ml
100ng/ml
AES16-2M
EGF

Figure S2

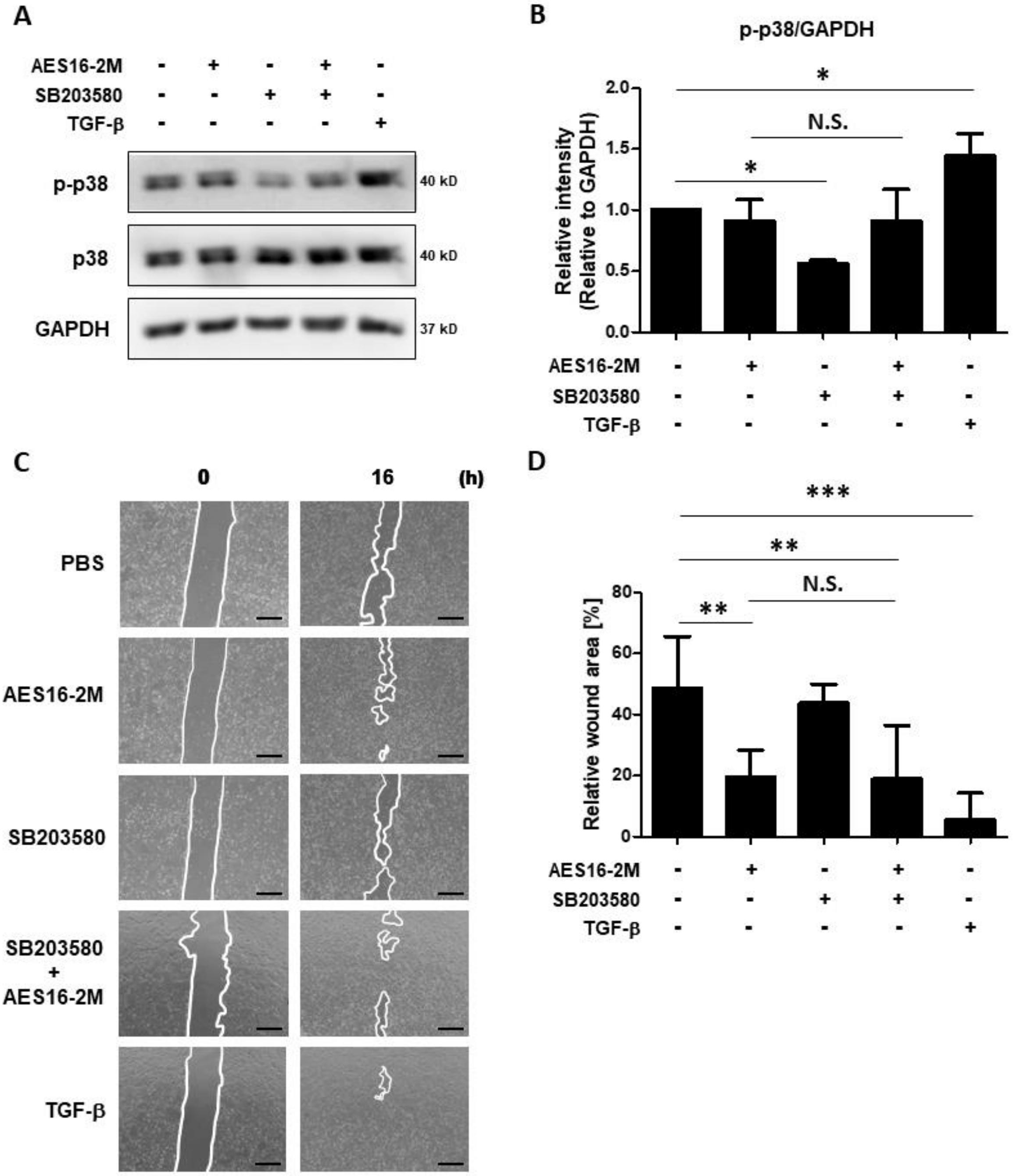
A
AES16-2M - + - + -
SB203580 - - + + -
TGF-β - - - - +
p-p38
40 kD
p38
40 kD
GAPDH
37 kD
B
p-p38/GAPDH
Relative intensity (Relative to GAPDH)
2.0
1.5
1.0
0.5
0.0
*
N.S.
*
AES16-2M - + - + -
SB203580 - - + + -
TGF-β - - - - +
C
0
16
(h)
PBS
AES16-2M
SB203580
SB203580 + AES16-2M
TGF-β
D
Relative wound area [%]
80
60
40
20
0
***
**
N.S.
**
AES16-2M - + - + -
SB203580 - - + + -
TGF-β - - - - +

Figure S3

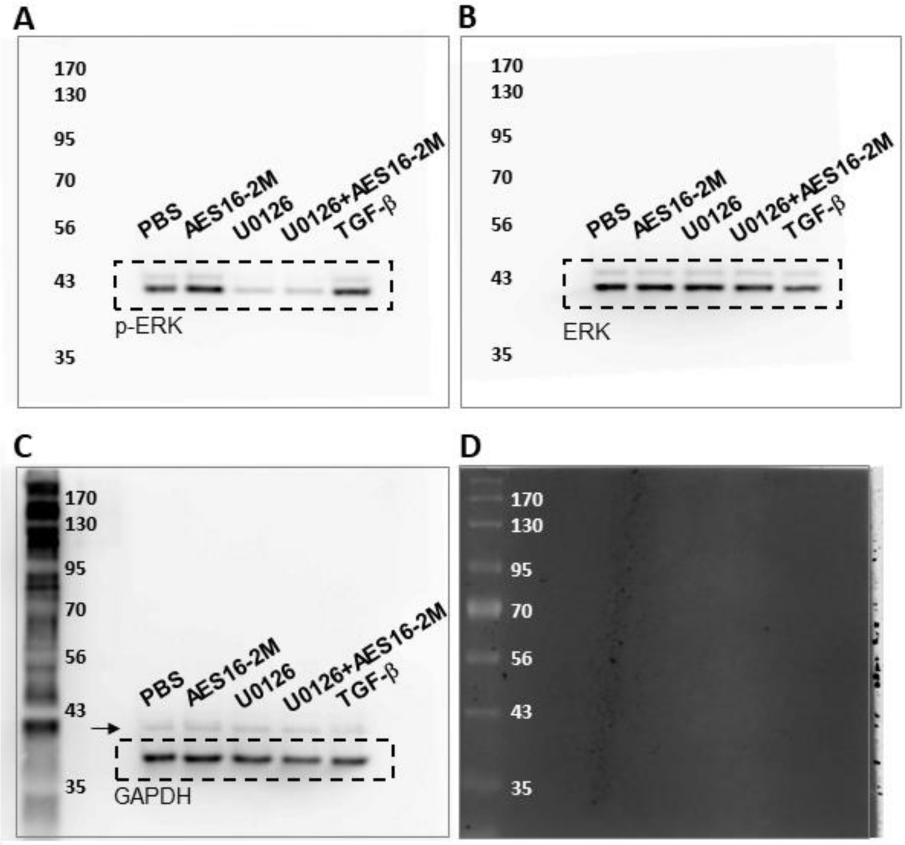
A
170
130
95
70
56
43
35
PBS
AES16-2M
U0126
U0126+AES16-2M
TGF-β
p-ERK
B
170
130
95
70
56
43
35
PBS
AES16-2M
U0126
U0126+AES16-2M
TGF-β
ERK
C
170
130
95
70
56
43
35
PBS
AES16-2M
U0126
U0126+AES16-2M
TGF-β
GAPDH
D
170
130
95
70
56
43
35

Figure S4

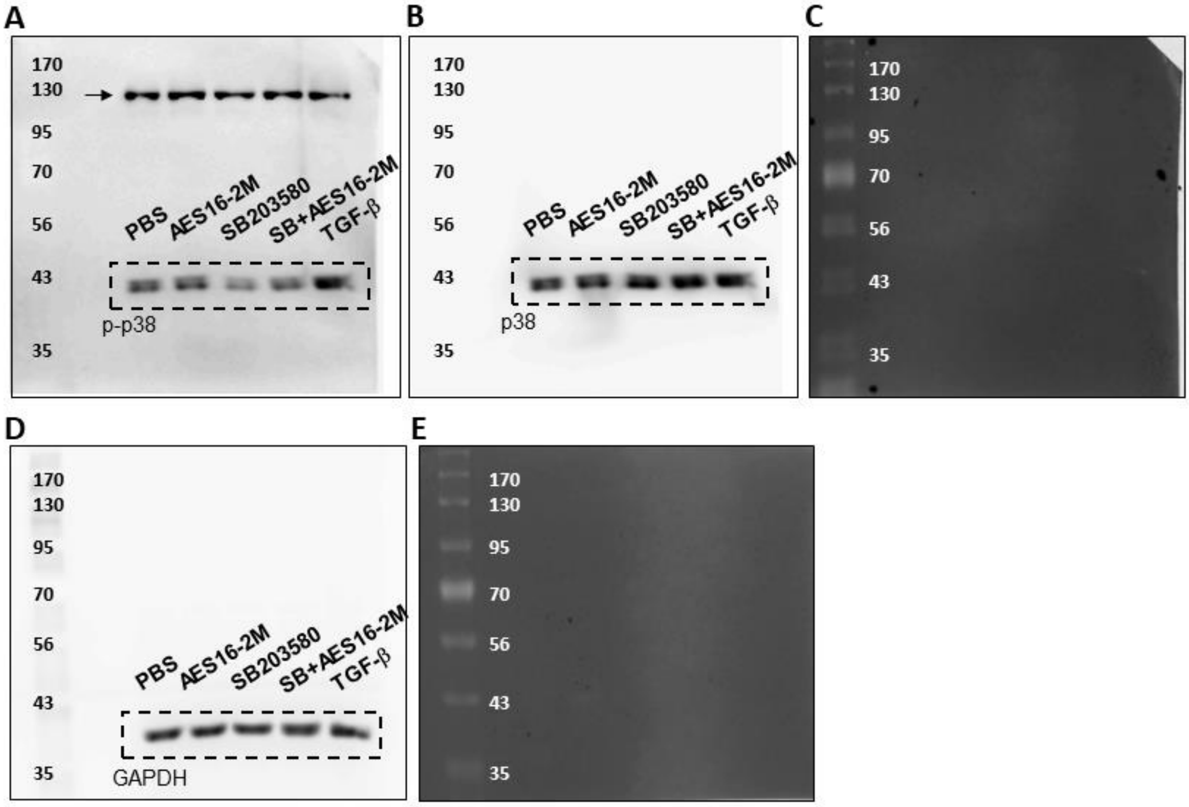
A
170
130
95
70
56
43
35
PBS
AES16-2M
SB203580
SB+AES16-2M
TGF-β
p-p38
B
170
130
95
70
56
43
35
PBS
AES16-2M
SB203580
SB+AES16-2M
TGF-β
p38
C
170
130
95
70
56
43
35
D
170
130
95
70
56
43
35
PBS
AES16-2M
SB203580
SB+AES16-2M
TGF-β
GAPDH
E
170
130
95
70
56
43
35
